# Emergence of Carbapenem-Resistant Uropathogenic *Escherichia coli* (ST405 and ST167) Strains Carrying *bla*_CTX-M-15_, *bla*_NDM-5_ and Diverse Virulence Factors in Hospitalized Patients

**DOI:** 10.3390/pathogens13110964

**Published:** 2024-11-05

**Authors:** Fatima Mujahid, Muhammad Hidayat Rasool, Muhammad Shafiq, Bilal Aslam, Mohsin Khurshid

**Affiliations:** 1 Institute of Microbiology, Government College University Faisalabad, Faisalabad 38000, Pakistan; fatimaajamal376@gmail.com (F.M.); mohsinkhurshid@gcuf.edu.pk (M.K.); 2 Research Institute of Clinical Pharmacy, Department of Pharmacology, Shantou University Medical College, Shantou 515041, China

**Keywords:** carbapenems, virulence, *E. coli*, UTIs, ARGs, hospital infections, antibiotic stewardship

## Abstract

Background: Urinary tract infections (UTIs) are common infectious diseases in hospital settings, and they are frequently caused by uropathogenic *Escherichia coli* (UPEC). The emergence of carbapenem-resistant (Carb-R) *E. coli* strains poses a significant threat due to their multidrug resistance and virulence. This study aims to characterize the antimicrobial resistance and virulence profiles of Carb-R UPEC strains isolated from hospitalized patients. Methods: A total of 1100 urine samples were collected from patients in Lahore and Faisalabad, Pakistan, between May 2023 and April 2024. The samples were processed to isolate and identify *E. coli* using standard microbiological techniques and VITEK®2, followed by amplification of the *uid*A gene. Antimicrobial susceptibility was evaluated using the Kirby–Bauer disc diffusion method and broth microdilution. Resistance and virulence genes were detected through PCR and DNA sequencing, and sequence typing was performed using MLST. Results: Among the 118 Carb-R UPEC isolates, resistance was most frequently observed against sulfamethoxazole-trimethoprim (96.6%) and doxycycline (96.6%). All of the isolates remained sensitive to colistin and tigecycline. Sequence types ST405 (35.6%) and ST167 (21.2%) were predominant and carried the *bla*_CTX-M-15_ and *bla*_NDM-5_ genes. The distribution of virulence genes and a variety of antimicrobial resistance genes (ARGs), conferring resistance to aminoglycosides, fluoroquinolones, tetracyclines, and sulfonamides, were observed as specifically linked to certain sequence types. Conclusions: This study provides insights into the molecular epidemiology of carbapenem-resistant Uropathogenic *E. coli* (Carb-R UPEC) strains and highlights the presence of globally high-risk *E. coli* clones exhibiting extensive drug resistance phenotypes in Pakistani hospitals. The findings underscore the urgent need for enhanced surveillance and stringent antibiotic stewardship to manage the spread of these highly resistant and virulent strains within hospital settings.

## 1. Introduction

Urinary tract infection (UTI) is a prevalent infectious disease in both community and hospital settings, affecting individuals of all ages [1,2]. Uropathogenic *Escherichia coli* (UPEC) is recognized as the primary causative agent of UTIs. UPEC has the capability to colonize the human gastrointestinal tract and, under favorable conditions, can infect and establish colonization in the urinogenital tract [3]. The virulence factors and host-related characteristics of UPEC facilitate the development of UTIs. Various virulence factors, including fimbriae with adhesin tips, protectins, toxins, and iron-acquisition systems, have been identified as contributing to UPEC pathogenesis by promoting colonization and infection of the urethra [4,5]. Hospital-acquired UTIs caused by *E. coli* are a significant concern in healthcare settings. These infections are often associated with invasive procedures, prolonged hospital stays, and the presence of urinary catheters. *E. coli* strains causing hospital-acquired UTIs can exhibit resistance to multiple antibiotics, posing challenges in treatment [2,6]. Surveillance, prevention, and appropriate antibiotic selection based on regional susceptibility data are crucial for effectively managing these infections.

Certain high-risk pandemic sequence types of *E. coli*, notably ST131, ST648, ST38, ST405, ST1193, ST410, and ST10, are increasingly prevalent in hospital settings, posing a significant public health threat [7,8,9,10]. These clones exhibit high genetic diversity, possess a broad spectrum of virulence factors, and demonstrate multi-drug resistance, enabling them to effectively transmit and cause infections in both community and healthcare environments. Many of these clones produce extended-spectrum beta-lactamases (ESBLs) and other resistance mechanisms, rendering them resistant to critical antibiotics such as carbapenems and third-generation cephalosporins. Studies indicate that these high-risk *E. coli* clones are frequently isolated from urinary tract infections, bloodstream infections, and other extraintestinal infections, are they are often associated with high mortality rates, particularly in resource-limited countries. They have also been identified in domestic animals and birds, further complicating efforts to control their spread and potential transmission between humans and animals [11]. *E. coli* sequence type (ST) 167 is globally recognized as the predominant ST among extraintestinal pathogenic *E. coli* (ExPEC) and is frequently linked to carbapenem resistance [12]. Studies have demonstrated that Carb-R *E. coli* ST167 strains carrying the *bla*_NDM-5_ gene can infect both humans and animals [13]. Notably, *E. coli* ST167 carrying the *bla*_NDM-5_ gene has been detected in freshwater fish in India [14]. Similarly, ST405 *E. coli* is identified as the most common Carb-R sequence type, exhibiting a worldwide distribution and representing a multidrug-resistant uropathogenic clone [15,16].

In Pakistan, ST405 (44.4%) and ST131 (29.2%) were identified as the most frequent sequence types among 184 Carb-R clinical strains of *E. coli* obtained from clinical specimens in Lahore [17]. Another study conducted in the same year reported a 14.4% prevalence of Carb-R Enterobacteriaceae (CPE) from 306 rectal swabs collected from patients at a tertiary care hospital in Rawalpindi, Pakistan, with ST167 and ST405 identified as the dominant sequence types [18]. Despite the increasing recognition of Carb-R *E. coli* strains, particularly ST405 and ST167, there is a gap in studies investigating their involvement in urinary tract infections (UTIs), especially healthcare-associated UTIs. Furthermore, while studies have highlighted the genetic diversity and virulence potential of these strains, there remains a lack of detailed investigations into the specific virulence factors contributing to their pathogenicity. This study aims to characterize the antimicrobial resistance patterns and virulence attributes of Carb-R uropathogenic *E. coli* (UPEC) strains isolated from hospitalized patients and to enhance understanding of the molecular epidemiology of these strains.

## 2. Materials and Methods

### 2.1. Ethical Approval

The research proposal was approved by the Ethical Review Committee (ERC), Government College University, Faisalabad, Pakistan, with reference number GCUF/ERC/23/16.

### 2.2. Specimen Collection and Bacterial Identification

A total of 1100 urine samples were collected from the suspected UTI patients admitted to three different tertiary care hospitals of Lahore and Faisalabad from May 2023 to April 2024. Only samples obtained from patients who had been admitted for 48 h or more were included in this study; samples collected from patients with symptoms at the time of admission or within the first 48 h were excluded. Urine samples were obtained based on the assessment of patient symptoms by clinicians as a part of routine diagnostic procedures performed at the healthcare facility. The samples were immediately transported to the laboratory and were inoculated on cystine lactose electrolyte deficient (CLED) agar (Oxoid, UK) plates using sterilized wire loop (nichrome) with an internal diameter of 4 mm holding 0.01 mL of urine sample. The plates were incubated at 37 °C and were observed for growth after 24 h. All isolates were further analyzed using the VITEK-2 compact system (bioMérieux, Marcy-l’Étoile, France) with the GN (Gram-negative) identification card. The VITEK-2 analysis was performed according to the manufacturer’s instructions.

### 2.3. Molecular Confirmation

For the molecular confirmation of *E. coli*, DNA was extracted using a DNA extraction kit (Favorgen Biotech Corporation, Taiwan, China). The purity of the DNA was assessed by measuring the absorbance at 260 and 280 nm using NanoDrop ™ (Thermo Fisher Scientific, Crawley, UK). The *E. coli*-specific *uid*A gene was amplified using PCR with the primers and annealing temperature detailed in Table S1. The amplicons were run on agarose gel electrophoresis and visualized using a UV transilluminator.

### 2.4. Antibiotic Susceptibility Pattern of CR-UPEC

The susceptibility of the *E. coli* isolates to various antimicrobial agents was assessed using both the disk diffusion method and the broth microdilution method following the Clinical and Laboratory Standards Institute (CLSI) 2023 guidelines.

The discs (Oxoid, UK) for the following antimicrobial agents were tested against the *E. coli* isolates: cefotaxime (CTX), ceftriaxone (CRO), fosfomycin (FOS), nitrofurantoin (F), imipenem (IPM), meropenem (MEM), amikacin (AK), gentamicin (CN), doxycycline (DO), and trimethoprim-sulfamethoxazole (SXT). For the disk diffusion method, sterile Mueller–Hinton agar plates were inoculated with bacterial suspensions adjusted to a 0.5 McFarland standard. Antibiotic discs were placed on the inoculated plates using a sterile disc dispenser, ensuring adequate spacing between discs to avoid overlapping zones of inhibition. Plates were incubated at 37 °C for 18 h. After incubation, the diameters of the zones of inhibition were measured in millimeters (mm) using a calibrated ruler. The results were interpreted according to the CLSI 2023 guidelines, with zone diameter breakpoints used to classify the isolates as susceptible, intermediate, or resistant.

In addition, the broth microdilution method was used to determine the minimal inhibitory concentrations (MICs) of a panel of antimicrobial agents including CTX, CRO, IPM, MEM, AK, CN, CIP, DO, CT, and TGC. For each antimicrobial agent, serial two-fold dilutions were prepared in cation-adjusted Mueller–Hinton broth in 96-well microtiter plates. The bacterial suspension was standardized to a final concentration of approximately 5 × 10^5^ CFU/mL, and 100 µL was added to each well. The plates were incubated at 37 °C for 16–20 h. The MIC was defined as the lowest concentration of the antibiotic that inhibited visible bacterial growth. Growth was visually assessed or using a spectrophotometer to measure optical density. The CLSI 2023 guidelines were followed to interpret the results, except for tigecycline for which the US FDA interpretive criteria were used: MIC ≤ 2 µg/mL (susceptible), MIC = 4 µg/mL (intermediate), and MIC ≥ 8 µg/mL (resistant). *E. coli* (ATCC^®^ 25922) and *Pseudomonas aeruginosa* (ATCC^®^ 27853) were used for quality control.

### 2.5. Screening for Antimicrobial Resistance Determinants in UPEC

Carb-R *E. coli* isolates were screened for the presence of antimicrobial resistance genes. All Carb-R *E. coli* isolates were screened for ESBL-encoding genes, including *bla*_CTX-M_, *bla*_TEM_, and *bla*_SHV_, using specific primers (Table S1). Additionally, these isolates were screened for *bla*_CTX-M_ variants including *bla*_CTX-M-1_, *bla*_CTX-M-2_, *bla*_CTX-M-8_, *bla*_CTX-M-9_, *bla*_CTX-M-10_, *bla*_CTX-M-14_, and *bla*_CTX-M-15_. To identify carbapenemase genes, isolates were screened for *bla*_IMP_, *bla*_VIM_, *bla*_NDM_, *bla*_SPM_, *bla*_GIM_, *bla*_SIM_, *bla*_KPC_, and *bla*_OXA-48_. The entire *bla*_NDM_ gene was amplified using the primers listed in Table S1 to determine the *bla*_NDM_ variants, and these were then subsequently sequenced. Furthermore, isolates were screened for plasmid-mediated quinolone resistance genes (PMQRs), including *qep*A, *qnr*A, *qnr*B, and *qnr*S; sulfonamide resistance genes (*sul*1 and *sul*2); aminoglycoside modifying enzymes (AMEs) (*aac(6*′*)-Ib*, *aph(3*″*)-Ib*, and *ant(2*″*)-Ia*); 16S methylases (*arm*A, *rmt*A, *rmt*B, *rmt*C, *rmt*D, *rmt*E, and *rmt*F); and tetracycline resistance genes (*tet*A and *tetB*).

### 2.6. Virulence Profiling of CR-UPEC Isolates

All Carb-R *E. coli* isolates were screened for the presence of virulence genes encoding adhesins (*fim*H, *pap*C, and *pap*G), iron acquisition (*fyu*A, *iut*A, and *irp*2), immune evasion (*tra*T and *cap*U), and tissue invasion (*hly*A and *KpsMTII*) using specific primers and annealing temperature (Table S1). The PCR products were separated by agarose gel electrophoresis (1.5% *w*/*v*) using a 100 bp DNA ladder as a molecular weight marker and were visualized under UV trans-illumination.

### 2.7. Multi-Locus Sequence Typing (MLST)

Multi-locus sequence typing (MLST) was performed on all Carb-R *E. coli* isolates targeting seven housekeeping genes (*adk, fumC, gyrB, icd, mdh, purA*, and *recA*). PCR amplification of these genes was followed by Sanger sequencing through Macrogen (Seoul, Republic of Korea). The obtained sequences were analyzed using the *E. coli* MLST database. Sequence assembly and alignment were conducted using ChromasPro version 2.1.8 and ClustalW version 2.0 respectively. Each gene locus was assigned an allelic number based on the *E. coli* MLST database, and the corresponding allelic profiles were used to determine the sequence types (STs) via the EnteroBase database.

### 2.8. Statistical Analysis

The data obtained were recorded in a Microsoft Excel 365 spreadsheet. Initial statistical analysis, including calculation of frequencies and percentage, was performed using Microsoft Excel. SPSS Statistics version 27.0 (IBM) was used to perform chi-square tests to compare the prevalence of the resistant phenotypes, resistance genes, and virulence factors among different hospitals and sequence types of *E. coli*. A *p*-value of less than 0.05 was considered statistically significant. However, since the chi-square test was used repeatedly for multiple analyses, the Bonferroni correction was applied, and the threshold for statistical significance was set at *p* < 0.001.

## 3. Results

In this study, a total of 1100 urine samples were received in our laboratory from three different tertiary care hospitals. Of these, 657 samples (59.7%) tested positive for bacterial or Candida growth. *Escherichia coli* (339 isolates, 51.6%) was the most prevalent pathogen associated with UTIs among the hospitalized patients. Of the *E. coli* isolates, 118 (34.8%) were found to be resistant to carbapenems.

### 3.1. Antimicrobial Resistance

**Figure 1:**
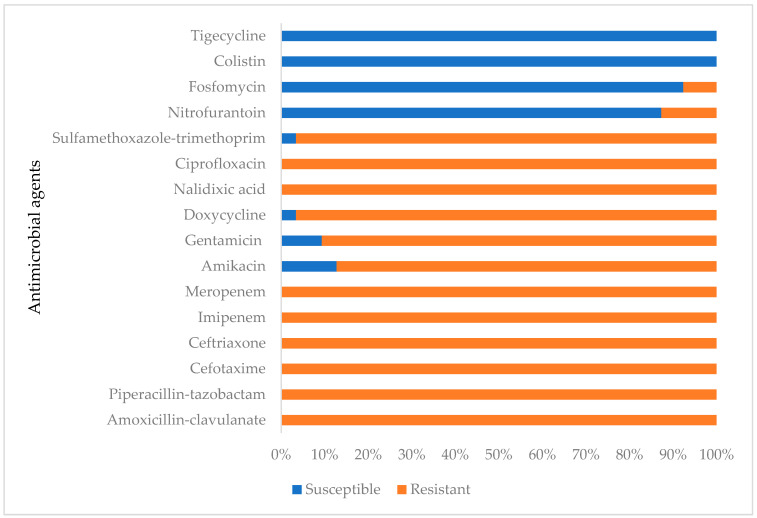
Antimicrobial resistance patterns of the Carb-R UPEC strains.

**Figure 2:**
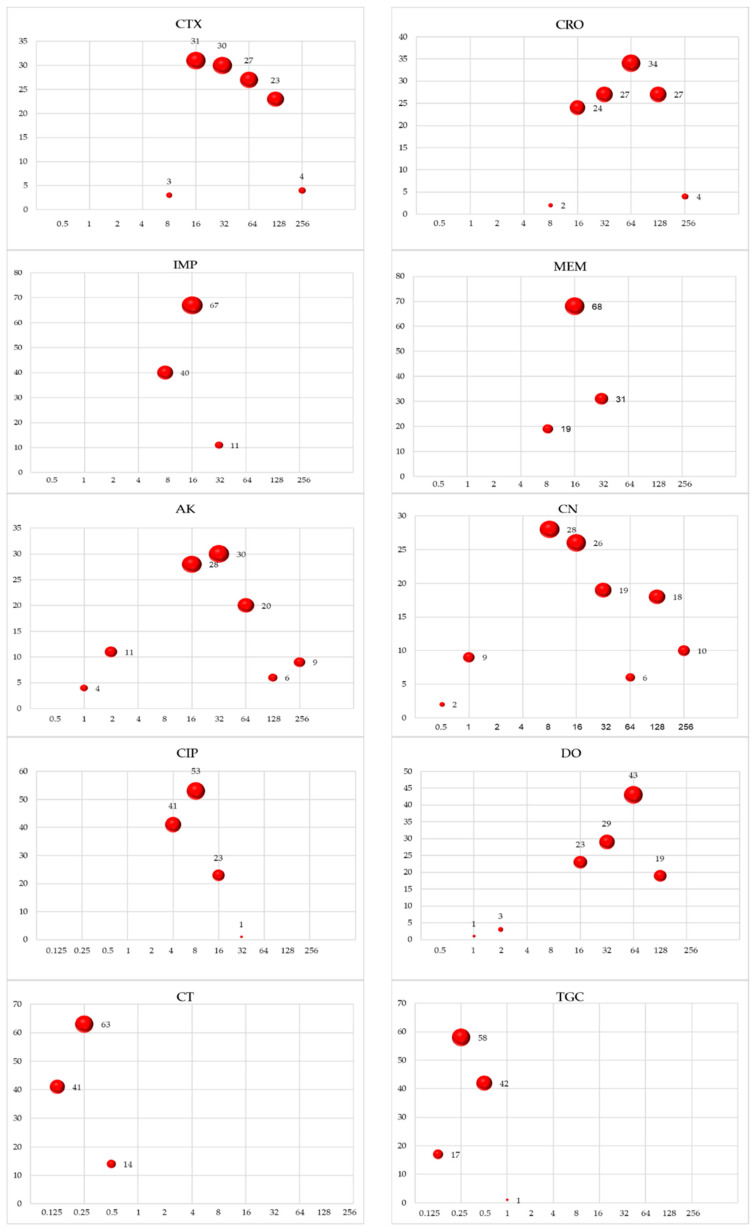
Bubble plots of the minimum inhibitory concentration distribution to various antimicrobial agents against Carb-R UPEC.

The Carb-R UPEC strains exhibited variable levels of resistance to the antibiotics. The highest resistance was observed against sulfamethoxazole-trimethoprim and doxycycline (96.6%, 114/118). This was followed by amikacin (87.3%, 103/118) and gentamicin (90.7%, 107/118). In contrast, resistance was lower for nitrofurantoin (12.7%, 15/118) and fosfomycin (7.6%, 9/118). All of the isolates were resistant to amoxicillin-clavulanate, piperacillin-tazobactam, cefotaxime, and ceftriaxone in addition to the carbapenems. However, all of the tested isolates remained susceptible to colistin and tigecycline (Figure 1 and Figure 2).

Although variations in resistance were observed among the hospitals, statistically significant resistance was found for fosfomycin (*p*-value ≤ 0.05). Specifically, only 9 out of 26 isolates (34.6%) from a hospital in Faisalabad, Pakistan, were resistant to fosfomycin, whereas isolates from the two hospitals in Lahore, Pakistan, were all susceptible to this antibiotic (Table 1).

#### Characterization of Antibiotic Resistance Genes (ARGs)

The prevalence of ARGs among carbapenem-resistant UPEC isolates revealed that, within the ESBLs, the *bla*_CTX-M_ genes were the most prevalent. Specifically, the *bla*_CTX-M-15_ gene was identified in 97 isolates (82.2%), making it the most common ESBL. This was followed by the *bla*_TEM_ gene, which was present in 39.8% of the isolates. Regarding carbapenemases, the *bla*_NDM_ gene was the sole metallo-beta-lactamase (MBL) detected, appearing in all isolates. Among these, the *bla*_NDM-5_ gene was more frequent, occurring in 86 isolates (72.9%), while the *bla*_NDM-1_ gene was found in 32 isolates (27.1%). Additionally, the *bla*_OXA-48_ gene, which encodes for oxacillinase, was detected in 14 isolates (11.9%). For aminoglycosides, aminoglycoside-modifying enzymes (AMEs) were predominant. The *aac(6*′*)-Ib* gene was found in 91 isolates (77.1%), while the *aph(3*′*)-Ib* gene was present in 89 isolates (75.4%). Among the 16S rRNA methylases, only the *rmt*B gene was detected, occurring in 22 isolates (18.6%). The *tet*B gene was the most common tetracycline resistance gene, detected in 90 isolates (76.3%), followed by *tet*A, which was found in 45 isolates (38.1%). Among the PMQRs, the *qep*A was present in 62 isolates (52.5%), whereas *qnr*B and *qnr*S were detected in 8 isolates (6.8%) and 10 isolates (8.5%), respectively, and no isolates were found to harbor the *qnr*A gene. Sulfonamide resistance was primarily associated with the *sul*1 gene, identified in 104 isolates (88.1%), while the *sul* 2 gene was present in 25 isolates (21.2%) (Table 1). The ARGs showed variable prevalence across the hospitals, with statistical significance being observed (*p*-value ≤ 0.05). Notably, the *bla*_SHV_, *bla*_OXA-48_, bla_NDM-1_, *bla*_NDM-5_, *ant(2*″*)-Ia*, *tet*A, *qnr*S, and *sul*1 genes exhibited significant variability in prevalence (Table 1).

### 3.2. Prevalence of Virulence Genes

The virulence genes showed varying prevalence among the Carb-R UPEC isolates across the three hospitals. The *fim*H gene was the most prevalent found in 96.6% of isolates, followed by *tra*T (93.2%) and *iut*A (89%). The *cap*U gene was the least prevalent (28.8%). Significant differences were observed in the prevalence of several genes: *hyl*A (*p* = 0.017), *fyu*A (*p* = 0.021), *pap*C (*p* < 0.001), *pap*G (*p* < 0.001), *cap*U (*p* = 0.001), and *kpsMTII* (*p* < 0.001), as shown in Table 1.

### 3.3. Sequence Types of the Carb-R EPEC Isolates

Among the 118 Carb-R UPEC isolates, the distribution of sequence types (STs) revealed notable patterns. The most common sequence type was ST405, comprising 42 isolates (35.6%), followed by ST167 with 25 isolates (21.2%) and ST10 with 11 isolates (9.3%).

The resistance patterns of 118 Carb-R *E. coli* isolates were evaluated across various sequence types (STs), revealing distinct profiles. The ST10 and ST1702 showed 100% susceptibility to amikacin, while all other STs were fully resistant (*p* < 0.001). Similarly, ST10 was 100% susceptible to gentamicin, with resistance observed in all other STs (*p* < 0.001). For doxycycline, ST410 isolates displayed complete susceptibility, whereas all other STs were resistant (*p* < 0.001). Two out of six of the isolates (33.3), corresponding to ST940, were resistant to sulfamethoxazole-trimethoprim, while all other STs showed 100% resistance (*p* < 0.001). Nitrofurantoin resistance was observed in ST10 (90.9% resistant) and ST101 (55.6% resistant), with other STs exhibiting 100% susceptibility (*p* < 0.001). Fosfomycin resistance was restricted to ST101 (100% resistant), with other STs showing susceptibility (*p* < 0.001) (Table 2).

The distribution of antibiotic resistance genes significantly varied among the different STs. For example, *bla*_SHV_ was present in ST10 (63.6%) and ST101 (88.9%) but was absent in other STs (*p* < 0.001). Similarly, *bla*_TEM_ was detected in ST10 and ST940 (100%) but was less common in other STs (*p* < 0.001); *bla*_CTX-M-15_ was identified in ST405, ST101, ST131, ST410, ST1702, and ST2851, with a significantly lower prevalence in ST167 and ST648 (*p* < 0.001); *bla*_OXA-48_ was only observed in ST167 and ST101 (*p* < 0.001); *bla*_NDM-1_ was found in ST10, ST101, ST131, and ST2851 (*p* < 0.001); whereas *bla*_NDM-5_ was present in all STs except ST101 (*p* < 0.001) (Table 2).

The distribution of virulence factors also significantly varied across different STs. The virulence factor *tra*T was found in ST405, ST167, ST10, ST101, ST940, ST648, ST410, ST1702, and ST2851, but was absent in ST131 (*p* < 0.001). Notably, *hyl*A was exclusively present in ST167, ST10, ST101, ST648, and ST1702, while *cap*U was only detected in ST10, ST101, ST940, ST410, and ST1702 (Table 2 and Table 3).

## 4. Discussion

Urinary tract infections (UTIs) are the most prevalent infections caused by Gram-negative bacteria, posing a significant challenge to healthcare systems. The presence of carbapenem-resistant (CR) Enterobacterales adds complexity to treatment strategies. *Escherichia coli* is an important pathogen in both clinical and veterinary settings due to several factors related to its pathogenicity, antimicrobial resistance, and environmental persistence [19,20]. The CR *E. coli* strains have emerged as one of the most problematic antimicrobial-resistant bacteria globally. Surveillance and research on the epidemiology and molecular mechanisms of CR *E. coli* are needed to understand the distribution of these resistant strains and to prevent their further spread [21]. It is interesting to note that certain sequence types of CR *E. coli* are increasingly prevalent worldwide. The rising incidence of these STs is alarming due to their potential for rapid dissemination and outbreak formation. STs possess the ability to acquire resistance genes through horizontal gene transfer. Additionally, many of these STs harbor virulence factors that augment their pathogenicity and survival in diverse ecological niches. Factors such as international travel, trade, and migration significantly contribute to the global dissemination of these resistant clones [16,22]. In Pakistan, there are limited data on the molecular epidemiology of *E. coli*, particularly UPEC strains. This knowledge gap poses a substantial challenge in comprehending and combating the spread of antibiotic-resistant strains within the region. This study aims to investigate antimicrobial susceptibility patterns, detect antimicrobial resistance determinants and virulence genes, and explore the molecular epidemiology of carbapenem-resistant uropathogenic *E. coli* (CR UPEC) strains isolated from hospitalized patients.

*Escherichia coli* isolates belonging to ST405 have been implicated in the dissemination of genes encoding extended-spectrum β-lactamases (ESBLs), primarily CTX-M enzymes, carbapenemases (i.e., *bla*_NDM_), and 16S methylases such as *arm*A and *rmt*B [23,24,25]. ST405 is increasingly recognized as a prevalent extraintestinal pathogenic *E. coli* clone associated with the global spread of ESBLs and carbapenemases. The reports from various parts of world have linked *E. coli* ST405 to several outbreaks, highlighting its significant public health threat [26,27,28]. In our study, ST405 was identified as the most prevalent sequence type, accounting for 42 (35.6%) of the uropathogenic *E. coli* (UPEC) isolates. Interestingly, all ST405 isolates carried *bla*_CTX-M-15_, *bla*_NDM-5_, *qep*A, and sul1 genes, while none harbored 16S methylases. However, the prevalence of other ESBLs, as well as tetracycline resistance genes (*tet*A and *tet*B) and aminoglycoside resistance genes (AMEs), varied among the isolates. ST405 has been increasingly associated with the carriage of *bla*_NDM_ genes, particularly *bla*_NDM-5_, which poses a serious challenge in clinical settings due to its ability to confer resistance to a broad range of beta-lactam antibiotics [29]. In a study from Italy, the isolate belonging to ST405 harboring *bla*_NDM-5_ gene was resistant to β-lactam antibiotics, carbapenems, and ciprofloxacin; however, it was susceptible to fosfomycin, amikacin, colistin, and tigecycline [30]. *E. coli* ST405 usually harbors multiple antibiotic resistance genes, as well as virulence factors, which enhance the survival of strain and adaptability in various environments, facilitating its spread across different regions and populations. Clinical settings such as hospitals provide a conducive environment to the spread of ST405 due to the concentration of antibiotic use and the presence of vulnerable populations. Outbreaks of ST405 have been reported in various parts of the world, highlighting its ability to cause infections in healthcare settings [29,31].

During the past few years, ST167 has also emerged as the most prevalent ST among extraintestinal pathogenic *E. coli* worldwide. It has often been linked to carbapenem resistance [12]. Studies have shown that carbapenem-resistant *E. coli* ST167 strains carrying the blaNDM-5 gene can infect both humans and animals [13]. In a report from Switzerland, the clinical isolate of *E. coli* belonging to ST167 was recovered from a hospitalized patient, which produced *bla*_NDM-35_ and harbored *sul*1, *aad*A2, and *tet*(B) [32]. However, in the majority of studies, *E. coli* ST167 has been found to harbor *bla*_NDM-5_, as evidenced in various studies across different countries and regions globally [12,33,34,35,36].

ST10 is an important ST, and it is considered an extraintestinal pathogenic *E. coli* lineage. It is increasingly significant in human infections and has also been reported as coming from food and environmental sources. Previous studies have shown the predominance of ST10 in clinical and veterinary samples, including domestic and wild animals and poultry. Moreover, ST10 has been a predominant sequence type found in environmental samples in European and Asian countries [37,38]. The isolates corresponding to ST10 are known to carry diverse beta-lactamases genes, especially the *bla*_CTX-M_ variants, bla_NDM_, and the *bla*_OXA-48_ genes. [37]. In 2023, there was a surge in the occurrence of CR ST10 isolates, with reports from at least four continents, predominantly in Asia, North America, and Europe. Spain had the highest rate of ST10 isolates (34.90%), followed by Germany and France with an occurrence of 21.57% and 16.39%, respectively [10]. Moreover, it was reported that the ST10 strains lacked the *fyu*A gene [10]. This is consistent with the findings of this study as the ST10 isolates were found to harbor the *bla*_NDM-1_ gene and lacked the virulence gene *fyu*A.

*E. coli* ST131 has been described as a globally spread lineage, akin to a pandemic, with rapid dissemination across five continents. It contributes to the rising prevalence of multidrug-resistant (MDR) *E. coli* worldwide and is a frequent cause of human infections, particularly urinary tract infections (UTIs) [39]. However, not all *E. coli* ST131 isolates are resistant to carbapenems. Carbapenem resistance is emerging in some ST131 strains, making it an evolving phenomenon [31]. Despite this, carbapenems have been considered the treatment of choice for *E. coli* ST131 infections. In this study, ST131 was not the most common sequence type. However, it was found to harbor several notable genes, including *bla*_CTX-M-15_, *bla*_NDM-1_, and *qnr*B, and it exhibited resistance to β-lactam-β-lactamase inhibitor combinations (amoxicillin-clavulanate and piperacillin-tazobactam), third-generation cephalosporins, carbapenems, amikacin, gentamicin, tetracyclines, quinolones, fluoroquinolones, and co-trimoxazole. This may be attributed to the fact that we included only Carb-R *E. coli* strains for MLST rather than those that are susceptible to carbapenems and that not all ST131 isolates are resistant to carbapenems.

In the last decade, there has been a shift in the predominant sequence types from highly virulent ST131 and ST38 to more antibiotic-resistant types, such as ST410 and ST167 [10]. A survey of Carb-R Enterobacterales across 36 European countries has shown that *bla*_NDM-5_ is the most frequently reported carbapenemase in *Escherichia coli*. Additionally, ST405, along with ST167, ST10, and ST361, were the most prevalent clones in these regions [40]. These findings raise concerns and necessitate further investigations to confirm the extent of the spread and to describe the epidemiological and microbiological characteristics of the identified isolates. Certain Carb-R *E. coli* STs are increasingly dominant, primarily due to their carriage of multiple antibiotic resistance genes, such as *bla*_NDM_. Their ability to acquire and maintain resistance genes, their increased survival in diverse environments, and their effective transmission dynamics contribute to their dominance. This scenario poses a serious public health threat, potentially leading to higher rates of morbidity and mortality. Surveillance of these STs on a global scale is crucial to understanding their epidemiology and implementing effective control measures to mitigate their impact on public health.

While our study provides valuable insights into the genomic epidemiology of uropathogenic *E. coli* in our region, there are some limitations that present opportunities for future research. Firstly, we did not perform whole-genome sequencing (WGS) for a comprehensive phylogenetic analysis and could also uncover novel resistance determinants or structural variations. Secondly, our study focused on detecting the presence of virulence genes rather than quantifying their expression levels. An analysis of gene expression would offer functional insights into the pathogenicity of these strains, potentially revealing the differences in the virulence factor activity among isolates with similar genetic profiles. These additional analyses would significantly enhance our understanding of the molecular epidemiology and pathogenesis of UPEC in our region.

## 5. Conclusions

UTIs incur significant costs and diminish the quality of life of patients and may lead toward severe, life-threatening conditions such as urosepsis. The rise of carbapenem-resistant *E. coli* presents a major challenge, particularly in the context of UTIs. Understanding the genetic traits of *E. coli* strains associated with UTIs provides crucial insights into the evolution of AMR and the acquisition of virulence genes over time. Our study highlights the emergence and dominance of several STs linked to high levels of antimicrobial resistance. Notably, ST405, ST167, and ST10 are becoming increasingly prevalent, underscoring the complexity of managing resistant *E. coli* strains. In Pakistan, the lack of comprehensive data on the molecular epidemiology of UPEC strains underscores the urgent need for improved surveillance and research. This research offers valuable insights into the virulence and antimicrobial resistance genes and the molecular epidemiology of carbapenem-resistant UPEC strains. The shift from highly virulent to more resistant STs over the past decade highlights the evolving nature of these bacterial strains and the need for ongoing monitoring and intervention. Addressing this public health threat necessitates global collaboration to track the spread of these resistant clones and implement effective infection control measures to mitigate their impact on patient health and healthcare systems.

## Figures and Tables

**Table 1:** Hospital-wise distribution of antimicrobial resistance, resistance genes, and virulence genes among CR-UPEC.

Resistance Traits	Total Isolates *n* = 118 *n* (%)	Hospital A *n* = 59 *n* (%)	Hospital B *n* = 33 *n* (%)	Hospital C *n* = 26 *n* (%)	*p*-Value
Antimicrobial agents					
Amoxicillin-clavulanate	118 (100)	59 (100)	33 (100)	26 (100)	-
Piperacillin-tazobactam	118 (100)	59 (100)	33 (100)	26 (100)	-
Cefotaxime	118 (100)	59 (100)	33 (100)	26 (100)	-
Ceftriaxone	118 (100)	59 (100)	33 (100)	26 (100)	-
Imipenem	118 (100)	59 (100)	33 (100)	26 (100)	-
Meropenem	118 (100)	59 (100)	33 (100)	26 (100)	-
Amikacin	103 (87.3)	51 (86.4)	30 (90.9)	22 (84.6)	0.742
Gentamicin	107 (90.7)	51 (86.4)	30 (90.9)	26 (100)	0.14
Doxycycline	114 (96.6)	56 (94.9)	33 (100)	25 (96.2)	0.429
Nalidixic acid	118 (100)	59 (100)	33 (100)	26 (100)	-
Ciprofloxacin	118 (100)	59 (100)	33 (100)	26 (100)	-
Sulfamethoxazole-trimethoprim	114 (96.6)	59 (100)	30 (90.9)	25 (96.2)	0.069
Nitrofurantoin	15 (12.7)	7 (11.9)	3 (9.1)	5 (19.2)	0.491
Fosfomycin	9 (7.6)	-	-	9 (34.6)	<0.001
Colistin	-	-	-	-	-
Tigecycline	-	-	-	-	-
Resistance genes					
*bla*_SHV_	15 (12.7)	7 (11.9)	-	8 (30.8)	0.002
*bla*_TEM_	47 (39.8)	25 (42.4)	12 (36.4)	10 (38.5)	0.842
*bla*_CTXM__-1_	12 (10.2)	8 (13.6)	-	4 (15.4)	0.072
*bla*_CTXM__-15_	97 (82.2)	44 (74.6)	28 (84.8)	25 (96.2)	0.051
*bla*_OXA__-48_	14 (11.9)	3 (5.1)	-	11 (42.3)	<0.001
*bla*_NDM__-1_	32 (27.1)	13 (22.0)	6 (18.2)	13 (50)	0.011
*bla*_NDM__-5_	86 (72.9)	46 (78)	27 (81.8)	13 (50)	0.011
*aac(6*′*)-Ib*	91 (77.1)	45 (76.3)	25 (75.8)	21 (80.8)	0.88
*aph(3*″*)-Ib*	89 (75.4)	43 (72.9)	22 (66.7)	24 (92.3)	0.062
*ant(2*″*)-Ia*	9 (7.6)	-	-	9 (34.6)	<0.001
*rmt*B	22 (18.6)	12 (20.3)	7 (21.2)	3 (11.5)	0.571
*tet*A	45 (38.1)	22 (37.3)	7 (21.2)	16 (61.5)	0.007
*tet*B	90 (76.3)	42 (71.2)	28 (84.8)	20 (76.9)	0.334
*qnr*B	8 (6.8)	5 (8.5)	3 (9.1)	-	0.296
*qnr*S	10 (8.5)	9 (15.3)	-	1 (3.8)	0.026
*qep*A	62 (52.5)	29 (49.2)	18 (54.5)	15 (57.7)	0.74
*sul*1	104 (88.1)	58 (98.3)	30 (90.9)	16 (61.5)	<0.001
*sul*2	25 (21.2)	13 (22)	3 (9.1)	9 (34.6)	0.057
Virulence genes					
*tra*T	110 (93.2)	54 (91.5)	30 (90.9)	26 (100)	0.296
*iut*A	105 (89)	51 (86.4)	28 (84.8)	26 (100)	0.123
*irp*2	81 (68.6)	43 (72.9)	25 (75.8)	13 (50)	0.065
*hyl*A	54 (45.8)	25 (42.4)	11 (33.3)	18 (69.2)	0.017
*fyu*A	98 (83.1)	51 (86.4)	30 (90.9)	17 (65.4)	0.021
*pap*C	94 (79.7)	51 (86.4)	30 (90.9)	13 (50)	<0.001
*pap*G	80 (67.8)	45 (76.3)	27 (81.8)	8 (30.8)	<0.001
*cap*U	34 (28.8)	13 (22)	6 (18.2)	15 (57.7)	0.001
*fim*H	114 (96.6)	56 (94.9)	33 (100)	25 (96.2)	0.429
*kpsMTII*	93 (78.8)	51 (86.4)	30 (90.9)	12 (46.2)	<0.001

The *p*-values were calculated by comparing the occurrence of different traits among different hospitals.

**Table 2:** Distribution of resistance traits and virulence factors among various sequence types of CR-UPEC.

Resistance Traits	ST405 (n = 42)	ST167 (n = 25)	ST10 (n = 11)	ST101 (n = 9)	ST131 (*n* = 8)	ST940 (*n* = 6)	ST648 (*n* = 5)	ST410 (*n* = 4)	ST1702 (*n* = 4)	ST2851 (*n* = 4)	*p*-Value
	*n* (%)	*n* (%)	*n* (%)	*n* (%)	*n* (%)	*n* (%)	*n* (%)	*n* (%)	*n* (%)	*n* (%)	
Amoxicillin-clavulanate	42 (100)	25 (100)	11 (100)	9 (100)	8 (100)	6 (100)	5 (100)	4 (100)	4 (100)	4 (100)	-
Piperacillin-tazobactam	42 (100)	25 (100)	11 (100)	9 (100)	8 (100)	6 (100)	5 (100)	4 (100)	4 (100)	4 (100)	-
Cefotaxime	42 (100)	25 (100)	11 (100)	9 (100)	8 (100)	6 (100)	5 (100)	4 (100)	4 (100)	4 (100)	-
Ceftriaxone	42 (100)	25 (100)	11 (100)	9 (100)	8 (100)	6 (100)	5 (100)	4 (100)	4 (100)	4 (100)	-
Imipenem	42 (100)	25 (100)	11 (100)	9 (100)	8 (100)	6 (100)	5 (100)	4 (100)	4 (100)	4 (100)	-
Meropenem	42 (100)	25 (100)	11 (100)	9 (100)	8 (100)	6 (100)	5 (100)	4 (100)	4 (100)	4 (100)	-
Amikacin	42 (100)	25 (100)	-	9 (100)	8 (100)	6 (100)	5 (100)	4 (100)	-	4 (100)	<0.001
Gentamicin	42 (100)	25 (100)	-	9 (100)	8 (100)	6 (100)	5 (100)	4 (100)	4 (100)	4 (100)	<0.001
Doxycycline	42 (100)	25 (100)	11 (100)	9 (100)	8 (100)	6 (100)	5 (100)	-	4 (100)	4 (100)	<0.001
Nalidixic acid	42 (100)	25 (100)	11 (100)	9 (100)	8 (100)	6 (100)	5 (100)	4 (100)	4 (100)	4 (100)	-
Ciprofloxacin	42 (100)	25 (100)	11 (100)	9 (100)	8 (100)	6 (100)	5 (100)	4 (100)	4 (100)	4 (100)	-
Sulfamethoxazole-trimethoprim	42 (100)	25 (100)	11 (100)	9 (100)	8 (100)	2 (33.3)	5 (100)	4 (100)	4 (100)	4 (100)	<0.001
Nitrofurantoin	-	-	10 (90.9)	5 (55.6)	-	-	-	-	-	-	<0.001
Fosfomycin	-	-	-	9 (100)	-	-	-	-	-	-	<0.001
Colistin	-	-	-	-	-	-	-	-	-	-	-
Tigecycline	-	-	-	-	-	-	-	-	-	-	-
Resistance genes											
*bla*_SHV_	-	-	7 (63.6)	8 (88.9)	-	-	-	-	-	-	<0.001
*bla*_TEM_	6 (14.3)	4 (16)	11 (100.0)	6 (66.7)	5 (62.5)	6 (100)	5 (100)	4 (100)	-	-	<0.001
*bla*_CTXM__-1_	-	-	8 (72.7)	-	-	-	-	-	4 (100)	-	<0.001
*bla*_CTXM-15_	42 (100)	21 (84)	3 (27.3)	9 (100)	8 (100)	-	2 (40)	4 (100)	4 (100)	4 (100)	<0.001
*bla*_OXA__-48_	-	4 (16)	1 (9.1)	9 (100)	-	-	-	-	-	-	<0.001
*bla*_NDM__-1_	-	-	11 (100)	9 (100)	8 (100)	-	-	-	4 (100)	-	<0.001
*bla*_NDM__-5_	42 (100)	25 (100)	-	-	-	6 (100)	5 (100)	4 (100)	-	4 (100)	<0.001
*aac(6*′*)-Ib*	42 (100)	25 (100)	-	9 (100)	8 (100)	6 (100)	-	-	-	1 (25)	<0.001
*aph(3*″*)-Ib*	42 (100)	25 (100)	-	9 (100)	8 (100)	-	-	-	4 (100)	1 (25)	<0.001
*ant(2*″*)-Ia*	-	-	-	9 (100)	-	-	-	-	-	-	<0.001
*rmt*B	10 (23.8)	-	-	-	-	-	5 (100)	4 (100)	-	3 (75)	<0.001
*tet*A	16 (38.1)	14 (56)	-	9 (100)	-	-	2 (40)	-	-	4 (100)	<0.001
*tet*B	36 (85.7)	11 (44)	11 (100)	9 (100)	8 (100)	6 (100)	5 (100)	-	4 (100)	-	<0.001
*qnr*B	-	-	-	-	8 (100)	-	-	-	-	-	<0.001
*qnr*S	-	4 (16)	-	-	-	2 (33.3)	-	4 (100)	-	-	<0.001
*qep*A	42 (100)	11 (44)	-	9 (100)	-	-	-	-	-	-	<0.001
*sul*1	42 (100)	25 (100)	10 (90.9)	-	8 (100)	2 (33.3)	5 (100)	4 (100)	4 (100)	4 (100)	<0.001
*sul*2	-	-	8 (72.7)	9 (100)	8 (100)	-	-	-	-	-	<0.001
Virulence factors											
*tra*T	42 (100)	25 (100)	11 (100)	9 (100)	-	6 (100)	5 (100)	4 (100)	4 (100)	4 (100)	<0.001
*iut*A	42 (100)	25 (100)	11 (100)	9 (100)	-	6 (100)	-	4 (100)	4 (100)	4 (100)	<0.001
*irp*2	42 (100)	25 (100)	-	-	-	6 (100)	-	4 (100)	-	4 (100)	<0.001
*shyl*A	-	25 (100)	11 (100)	9 (100)	-	-	5 (100)	-	4 (100)	-	<0.001
*fyu*A	42 (100)	25 (100)	-	-	-	6 (100)	5 (100)	4 (100)	4 (100)	4 (100)	<0.001
*pap*C	42 (100)	25 (100)	-	-	8 (100)	6 (100)	5 (100)	4 (100)	-	4 (100)	<0.001
*pap*G	42 (100)	-	11 (100)	-	8 (100)	6 (100)	5 (100)	4 (100)	-	4 (100)	<0.001
*cap*U	-	-	11 (100)	9 (100)	-	6 (100)	-	4 (100)	4 (100)	-	<0.001
*fim*H	42 (100)	25 (100)	11 (100)	9 (100)	8 (100)	6 (100)	5 (100)	-	4 (100)	4 (100)	<0.001
*kpsMTII*	42 (100)	25 (100)	11 (100)	-	-	6 (100)	5 (100)	-	-	4 (100)	<0.001

The table correlates the different traits among sequence types. The *p*-values were calculated by comparing individual sequence types (STs) with each other. The percentage of STs was calculated with reference to the total number of STs, whereas the percentage of different traits was calculated with reference to the total number of isolates corresponding to each ST.

**Table 3:** Distribution of the sequence types with antimicrobial resistance genotypes and virulence genes.

STs	Co-Occurrence of Resistance Genes (n)	Antimicrobial Resistant Phenotype (n)	Co-Occurrence of Virulence Genes
ST405 (n = 42)	*bla*_TEM_, *bla*_CTX-M-15_, *bla*_NDM-5_, *aac(6*′*)-Ib*, *aph(3*″*)-Ib*, *tet*A, *qep*A, *sul*1 (06)	AMC, TZP, CTX, CRO, IPM, MEM, AK, CN, DO, NA, CIP, SXT (42)	*tra*T, *iut*A, *irp*2, *fyu*A, *pap*C, *pap*G, *fim*H, *kpsMTII*
	*bla*_CTX-M-15_, *bla*_NDM-5_, *aac(6*′*)-Ib*, *aph(3*″*)-Ib*, *rmt*B, *tet*A, *tet*B, *qep*A, *sul*1 (10)		
	*bla*_CTX-M-15_, *bla*_NDM-5_, *aac(6*′*)-Ib*, *aph(3*″*)-Ib*, *tet*B, *qep*A, *sul*1 (26)		
ST167 (n = 25)	*bla*_CTX-M-15_, *bla*_NDM-5_, *aac(6*′*)-Ib*, *aph(3*″*)-Ib*, *tet*B, *qep*A, *sul*1 (11)	AMC, TZP, CTX, CRO, IPM, MEM, AK, CN, DO, NA, CIP, SXT (25)	*tra*T*, iut*A, *irp*2, *hyl*A, *fyu*A, *pap*C, *fim*H, *kpsMTII*
	*bla*_CTX-M-15_, *bla*_NDM-5_, *aac(6*′*)-Ib*, *aph(3*″*)-Ib*, *tet*A, *sul*1 (06)		
	*bla*_TEM_, *bla*_CTX-M-15_, *bla*_OXA-48_, *bla*_NDM-5_, *aac(6*′*)-Ib*, *aph(3*″*)-Ib*, *tet*A, *sul*1 (04)		
	*bla*_NDM-5_, *aac(6*′*)-Ib*, *aph(3*″*)-Ib*, *tet*A, *qnr*S, *sul*1 (04)		
ST10 (n = 11)	*bla*_SHV_, *bla*_TEM_, *bla*_CTX-M-1_, *bla*_NDM-1_, *tet*B, *sul*1, *sul*2 (07)	AMC, TZP, CTX, CRO, IPM, MEM, DO, NA, CIP, SXT, F (10)	*tra*T, *iut*A, *hyl*A, *papG*, *cap*U, *fim*H, *kpsMTII*
	*bla*_TEM_, *bla*_CTX-M-15_, *bla*_NDM-1_, *tet*B, *sul*1 (03)		
	*bla*_TEM_, *bla*_CTX-M-1_, *bla*_OXA-48_, *bla*_NDM-1_, *tet*B, *sul*2 (01)	AMC, TZP, CTX, CRO, IPM, MEM, DO, NA, CIP, SXT (01)	
ST101 (n = 9)	*bla*_SHV_, *bla*_TEM_, *bla*_CTX-M-15_, *bla*_OXA-48_, *bla*_NDM-1_, *aac(6*′*)-Ib*, *aph(3*″*)-Ib*, *ant(2*″*)-Ia*, *tet*A, *tet*B, *qep*A, *sul*2 (05)	AMC, TZP, CTX, CRO, IPM, MEM, AK, CN, DO, NA, CIP, SXT, F, FOS (05)	*tra*T, *iut*A, *hyl*A, *cap*U, *FimH*
	*bla*_SHV_, *bla*_CTX-M-15_, *bla*_OXA-48_, *bla*_NDM-1_, *aac(6*′*)-Ib*, *aph(3*″*)-Ib*, *ant(2*″*)-Ia*, *tet*A, *tet*B, *qep*A, *sul*2 (03)	AMC, TZP, CTX, CRO, IPM, MEM, AK, CN, DO, NA, CIP, SXT, FOS (04)	
	*bla*_TEM_, *bla*_CTX-M-15_, *bla*_OXA-48_, *bla*_NDM-1_, *aac(6*′*)-Ib*, *aph(3*″*)-Ib*, *ant(2*″*)-Ia*, *tet*A, *tet*B, *qep*A, *sul*2 (01)		
ST131 (n = 8)	*bla*_TEM_, *bla*_CTX-M-15_, *bla*_NDM-1_, *aac(6*′*)-Ib*, *aph(3*″*)-Ib*, *tet*B, *qnr*B, *sul*1 (05)	AMC, TZP, CTX, CRO, IPM, MEM, AK, CN, DO, NA, CIP, SXT (08)	*fyu*A, *pap*C, *papG*, *capU*, *Fim*H
	*bla*_CTX-M-15_, *bla*_NDM-1_, *aac(6*′*)-Ib*, *aph(3*″*)-Ib*, *tet*B, *qnr*B, *sul*1, *sul*2 (03)		
ST940 (n = 6)	*bla*_TEM_, *aac(6*′*)-Ib*, *bla*_NDM-5_, *tet*B (04)	AMC, TZP, CTX, CRO, IPM, MEM, AK, CN, DO, NA, CIP (06)	*tra*T, *iut*A, *irp*2, *fyuA*, *pap*C, *papG*, *fim*H, *kpsMTII*
	*bla*_NDM-5_, *aac(6*′*)-Ib*, *tet*B,*qnr*S, *sul*1 (02)		
ST648 (n = 5)	*bla*_TEM_, *bla*_NDM-5_, *rmt*B, *tet*B, *sul*1 (03)	AMC, TZP, CTX, CRO, IPM, MEM, AK, CN, DO, NA, CIP, SXT (05)	*tra*T, *hyl*A, *fyuA*, *pap*C, *pap*G, *fim*H, *kpsMTII*
	*bla*_TEM_, *bla*_CTX-M-15_, *bla*_NDM-5_, *rmt*B *tet*A, *tet*B, *sul*1 (02)		
ST410 (n = 4)	*bla*_CTX-M-15_, *bla*_NDM-5_, *rmt*B, *sul*1 (04)	AMC, TZP, CTX, CRO, IPM, MEM, AK, CN, NA, CIP, SXT (04)	*tra*T, *iut*A, *irp*2, *fyu*A, *pap*C, *pap*G, *cap*U
ST1702 (n = 4)	*bla*_CTX-M-1_, *bla*_CTX-M-15_, *bla*_NDM-1_, *aph(3*″*)-Ib*, *tet*B, *sul*1 (04)	AMC, TZP, CTX, CRO, IPM, MEM, CN, DO, NA, CIP, SXT (04)	*tra*T, *iut*A, *hyl*A, *fyuA*, *cap*U, *FimH*
ST2851 (n = 4)	*bla*_CTX-M-15_, *bla*_NDM-5_, *rmt*B, *tet*A, *sul*1 (03)	AMC, TZP, CTX, CRO, IPM, MEM, AK, CN, DO, NA, CIP, SXT (04)	*tra*T, *iut*A, *irp2*, *fyu*A, *pap*C, *pap*G, *fim*H*, kpsMTII*
	*bla*_CTX-M-15_, *bla*_NDM-5_, *aac(6′)-Ib*, *aph(3″)-Ib*, *tet*A, *sul*1 (01)		

## Data Availability

The data that support the findings of this study are available from the corresponding authors upon reasonable request.

## Supplementary Materials

The following supporting information can be downloaded at: https://www.mdpi.com/article/10.3390/pathogens13110964/s1, Table S1. Primers used in this study. Table S2. Demographics, collection dates, and hospital ward information of the UTI Patients.

## References

[1] Sohail M., Khurshid M., Saleem H.G., Javed H., Khan A.A. (2015). Characteristics and Antibiotic Resistance of Urinary Tract Pathogens Isolated from Punjab, Pakistan. Jundishapur J. Microbiol..

[2] Toval F., Köhler C.D., Vogel U., Wagenlehner F., Mellmann A., Fruth A., Schmidt M.A., Karch H., Bielaszewska M., Dobrindt U. (2014). Characterization of *Escherichia coli* isolates from hospital inpatients or outpatients with urinary tract infection. J. Clin. Microbiol..

[3] Abd El Ghany M., Sharaf H., Al-Agamy M.H., Shibl A., Hill-Cawthorne G.A., Hong P.Y. (2018). Genomic characterization of NDM-1 and 5, and OXA-181 carbapenemases in uropathogenic *Escherichia coli* isolates from Riyadh, Saudi Arabia. PLoS ONE.

[4] Terlizzi M.E., Gribaudo G., Maffei M.E. (2017). UroPathogenic *Escherichia coli* (UPEC) Infections: Virulence Factors, Bladder Responses, Antibiotic, and Non-antibiotic Antimicrobial Strategies. Front. Microbiol..

[5] Whelan S., Lucey B., Finn K. (2023). Uropathogenic *Escherichia coli* (UPEC)-Associated Urinary Tract Infections: The Molecular Basis for Challenges to Effective Treatment. Microorganisms.

[6] Lee D.S., Lee S.J., Choe H.S. (2018). Community-Acquired Urinary Tract Infection by *Escherichia coli* in the Era of Antibiotic Resistance. BioMed Res. Int..

[7] Byarugaba D.K., Erima B., Wokorach G., Alafi S., Kibuuka H., Mworozi E., Musinguzi A.K., Kiyengo J., Najjuka F., Wabwire-Mangen F. (2023). Resistome and virulome of high-risk pandemic clones of multidrug-resistant extra-intestinal pathogenic *Escherichia coli* (ExPEC) isolated from tertiary healthcare settings in Uganda. PLoS ONE.

[8] Pitout J.D.D., Peirano G., Chen L., DeVinney R., Matsumura Y. (2022). *Escherichia coli* ST1193: Following in the Footsteps of *E. coli* ST131. Antimicrob. Agents Chemother..

[9] García-Meniño I., García V., Lumbreras-Iglesias P., Fernández J., Mora A. (2024). Fluoroquinolone resistance in complicated urinary tract infections: Association with the increased occurrence and diversity of *Escherichia coli* of clonal complex 131, together with ST1193. Front. Cell. Infect. Microbiol..

[10] Huang J., Lv C., Li M., Rahman T., Chang Y.F., Guo X., Song Z., Zhao Y., Li Q., Ni P. (2024). Carbapenem-resistant *Escherichia coli* exhibit diverse spatiotemporal epidemiological characteristics across the globe. Commun. Biol..

[11] Pöntinen A.K., Gladstone R.A., Pesonen H., Pesonen M., Cléon F., Parcell B.J., Kallonen T., Simonsen G.S., Croucher N.J., McNally A. (2024). Modulation of multidrug-resistant clone success in *Escherichia coli* populations: A longitudinal, multi-country, genomic and antibiotic usage cohort study. Lancet Microbe.

[12] Garcia-Fernandez A., Villa L., Bibbolino G., Bressan A., Trancassini M., Pietropaolo V., Venditti M., Antonelli G., Carattoli A. (2020). Novel Insights and Features of the NDM-5-Producing *Escherichia coli* Sequence Type 167 High-Risk Clone. mSphere.

[13] Grönthal T., Österblad M., Eklund M., Jalava J., Nykäsenoja S., Pekkanen K., Rantala M. (2018). Sharing more than friendship—Transmission of NDM-5 ST167 and CTX-M-9 ST69 *Escherichia coli* between dogs and humans in a family, Finland, 2015. Euro Surveill.

[14] Dwivedi A., Kumar C.B., Kumar A., Soni M., Sahu V., Awasthi A., Rathore G. (2023). Molecular characterization of carbapenem resistant *E. coli* of fish origin reveals the dissemination of NDM-5 in freshwater aquaculture environment by the high risk clone ST167 and ST361. Environ. Sci. Pollut. Res. Int..

[15] Alghoribi M.F., Gibreel T.M., Farnham G., Al Johani S.M., Balkhy H.H., Upton M. (2015). Antibiotic-resistant ST38, ST131 and ST405 strains are the leading uropathogenic *Escherichia coli* clones in Riyadh, Saudi Arabia. J. Antimicrob. Chemother..

[16] Chowdhury P.R., McKinnon J., Liu M., Djordjevic S.P. (2018). Multidrug Resistant Uropathogenic *Escherichia coli* ST405 with a Novel, Composite IS26 Transposon in a Unique Chromosomal Location. Front. Microbiol..

[17] Gondal A.J., Choudhry N., Bukhari H., Rizvi Z., Yasmin N. (2022). Characterization of Genomic Diversity among Carbapenem-Resistant *Escherichia coli* Clinical Isolates and Antibacterial Efficacy of Silver Nanoparticles from Pakistan. Microorganisms.

[18] Habib A., Lo S., Villageois-Tran K., Petitjean M., Malik S.A., Armand-Lefèvre L., Ruppé E., Zahra R. (2022). Dissemination of carbapenemase-producing Enterobacterales in the community of Rawalpindi, Pakistan. PLoS ONE.

[19] Khan A.Y., Ahmad S.S., Avais M., Ashraf K. (2022). Molecular prevalence with associated risk factors and haemato-serum electrolyte analysis of *E. coli* O157: H7 in Canine pups with diarrhoea. Pak. Vet. J..

[20] Li X., Zhu X., Xue Y. (2023). Drug resistance and genetic relatedness of *Escherichia coli* from mink in Northeast China. Pak. Vet. J..

[21] Tian X., Zheng X., Sun Y., Fang R., Zhang S., Zhang X., Lin J., Cao J., Zhou T. (2020). Molecular Mechanisms and Epidemiology of Carbapenem-Resistant *Escherichia coli* Isolated from Chinese Patients During 2002–2017. Infect. Drug Resist..

[22] von Wintersdorff C.J., Penders J., van Niekerk J.M., Mills N.D., Majumder S., van Alphen L.B., Savelkoul P.H., Wolffs P.F. (2016). Dissemination of Antimicrobial Resistance in Microbial Ecosystems through Horizontal Gene Transfer. Front. Microbiol..

[23] Coque T.M., Novais A., Carattoli A., Poirel L., Pitout J., Peixe L., Baquero F., Cantón R., Nordmann P. (2008). Dissemination of clonally related *Escherichia coli* strains expressing extended-spectrum beta-lactamase CTX-M-15. Emerg. Infect. Dis..

[24] Tian G.B., Rivera J.I., Park Y.S., Johnson L.E., Hingwe A., Adams-Haduch J.M., Doi Y. (2011). Sequence type ST405 *Escherichia coli* isolate producing QepA1, CTX-M-15, and RmtB from Detroit, Michigan. Antimicrob. Agents Chemother..

[25] Shin J., Kim D.H., Ko K.S. (2011). Comparison of CTX-M-14- and CTX-M-15-producing *Escherichia coli* and *Klebsiella pneumoniae* isolates from patients with bacteremia. J. Infect..

[26] Li F., Ye K., Li X., Ye L., Guo L., Wang L., Yang J. (2021). Genetic characterization of Carbapenem-Resistant *Escherichia coli* from China, 2015–2017. BMC Microbiol..

[27] Peirano G., Pitout J.D.D. (2019). Extended-Spectrum β-Lactamase-Producing Enterobacteriaceae: Update on Molecular Epidemiology and Treatment Options. Drugs.

[28] Bitar I., Piazza A., Gaiarsa S., Villa L., Pedroni P., Oliva E., Nucleo E., Pagani L., Carattoli A., Migliavacca R. (2017). ST405 NDM-5 producing *Escherichia coli* in Northern Italy: The first two clinical cases. Clin. Microbiol. Infect..

[29] Sumbana J.J., Santona A., Fiamma M., Taviani E., Deligios M., Zimba T., Lucas G., Sacarlal J., Rubino S., Paglietti B. (2021). Extraintestinal Pathogenic *Escherichia coli* ST405 Isolate Coharboring blaNDM-5 and blaCTXM-15: A New Threat in Mozambique. Microb. Drug Resist..

[30] Corbellini S., Scaltriti E., Piccinelli G., Gurrieri F., Mascherpa M., Boroni G., Amolini C., Caruso A., De Francesco M.A. (2022). Genomic characterisation of *Escherichia coli* isolates co-producing NDM-5 and OXA-1 from hospitalised patients with invasive infections. J. Glob. Antimicrob. Resist..

[31] Slown S., Walas N., Amato H.K., Lloyd T., Varghese V., Bender M., Pandori M., Graham J. (2022). Clonal Lineages and Virulence Factors of Carbapenem Resistant *E. coli* in Alameda County, California, 2017–2019. Antibiotics.

[32] Poirel L., Ortiz de la Rosa J.M., Sakaoglu Z., Kusaksizoglu A., Sadek M., Nordmann P. (2022). NDM-35-Producing ST167 *Escherichia coli* Highly Resistant to β-Lactams Including Cefiderocol. Antimicrob. Agents Chemother..

[33] Peterhans S., Stevens M.J.A., Nüesch-Inderbinen M., Schmitt S., Stephan R., Zurfluh K. (2018). First report of a bla(NDM-5)-harbouring *Escherichia coli* ST167 isolated from a wound infection in a dog in Switzerland. J. Glob. Antimicrob. Resist..

[34] Xu L., Wang P., Cheng J., Qin S., Xie W. (2019). Characterization of a novel bla (NDM-5)-harboring IncFII plasmid and an mcr-1-bearing IncI2 plasmid in a single *Escherichia coli* ST167 clinical isolate. Infect. Drug Resist..

[35] Manyahi J., Moyo S.J., Kibwana U., Goodman R.N., Allman E., Hubbard A.T.M., Blomberg B., Langeland N., Roberts A.P. (2022). First identification of bla (NDM-5) producing *Escherichia coli* from neonates and a HIV infected adult in Tanzania. J. Med. Microbiol..

[36] Ragupathi N.K.D., Veeraraghavan B., Sethuvel D.P.M., Anandan S., Vasudevan K., Neeravi A.R., Daniel J.L.K., Sathyendra S., Iyadurai R., Mutreja A. (2020). First Indian report on genome-wide comparison of multidrug-resistant *Escherichia coli* from blood stream infections. PLoS ONE.

[37] Fuga B., Sellera F.P., Cerdeira L., Esposito F., Cardoso B., Fontana H., Moura Q., Cardenas-Arias A., Sano E., Ribas R.M. (2022). WHO Critical Priority *Escherichia coli* as One Health Challenge for a Post-Pandemic Scenario: Genomic Surveillance and Analysis of Current Trends in Brazil. Microbiol. Spectr..

[38] Bojesen A.M., Ahmed U., Skaarup H., Espinosa-Gongora C. (2022). Recurring outbreaks by the same *Escherichia coli* ST10 clone in a broiler unit during 18 months. Vet. Res..

[39] Kudinha T., Kong F. (2022). Possible step-up in prevalence for *Escherichia coli* ST131 from fecal to clinical isolates: Inferred virulence potential comparative studies within phylogenetic group B2. J. Biomed. Sci..

[40] Linkevicius M., Bonnin R.A., Alm E., Svartström O., Apfalter P., Hartl R., Hasman H., Roer L., Räisänen K., Dortet L. (2023). Rapid cross-border emergence of NDM-5-producing *Escherichia coli* in the European Union/European Economic Area, 2012 to June 2022. Eurosurveillance.

