# Supplementary material for: Emergence of Carbapenem-Resistant Uropathogenic Escherichia coli (ST405 and ST167) Strains Carrying blaCTX-M-15, blaNDM-5 and Diverse Virulence Factors in Hospitalized Patients
Source: Pathogens. 2024 Nov 5;13(11):964. doi: 10.3390/pathogens13110964 (PMC11597634; doi:10.3390/pathogens13110964)
Supplement: Supplementary file 1 [file pathogens-13-00964-s001.zip › pathogens-3194500-supplementary.pdf]

Table S1: Primers used in this study

Genes	Primers	Sequence (5′ to 3′)	Aimed Product (bp)	Annealing Temperature (°C)
uidA	uidA-F	CAACGAACTGAACTGGCAGA	162	50
	uidA-R	CATTACGCTGCGATGGAT		
*bla*TEM	TEM-F	TCAACATTTCCGTGTCG	860	56
	TEM-R	CTGACAGTTACCAATGCTTA		
*bla*SHV	SHV-F	ATGCGTTATATTCGCCTGTG	896	56
	SHV-R	AGATAAATCACCACAATGCGC		
*bla*CTX-M	CTXMU-F	ATGTGCAGYACCAGTAARGTKATGGC	593	52
	CTXMU-R	TGGGTRAARTARGTSACCAGAAYCAGCGG		
*bla*CTX-M-1	CTX-M-1-F	CCGTTTCCGCTATTACAAACCGTTG	944	56
	CTX-M-1-R	GGCCCATGGTTAAAAAATCACTGC		
*bla*CTX-M-2	CTX-M-2-F	ATGATGACTCACAGCATTCG	833	56
	CTX-M-2-R	TCCCGACGGCTTTCCGCGTT		
*bla*CTX-M-8	CTX-M-8-F	TTTGCCCGTGCGATTGG	368	50
	CTX-M-8-R	CGACTTTCTGCCTTCTGCTC		
*bla*CTX-M-9	CTX-M-9-F	ATGGTGACAAAGAGAGTGCA	870	50
	CTX-M-9-R	CCCTTCGGCGATGATTCTC		
*bla*CTX-M-10	CTX-M-10-F	GCAGCACCAGTAAAGTGATGG	524	56
	CTX-M-10-R	GCGATATCGTTGGTGGTACC		
*bla*CTX-M-14	CTX-M-14-F	GAGAGTGCAACGGATGATG	941	56
	CTX-M-14-R	TGCGGCTGGGTAAAATAG		
*bla*CTX-M-15	CTX-M-15-F	CACACGTGGAATTTAGGGACT	996	50
	CTX-M-15-R	GCCGTCTAAGGCGATAAACA		
*bla*OXA-48	OXA-48-F	TTGGTGGCATCGATTATCGG	744	52
	OXA-48-F	GAGCACTTCTTTTGTGATGGC		
*bla*IMP	IMP-F	GGAATAGAGTGGCTTAAYTC	232	50
	IMP-R	TCGGTTTAAYAAAACAACCACC		
*bla*VIM	VIM-F	GATGGTGTTTGGTCGCATA	390	50
	VIM-R	CGAATGCGCAGCACCAG		
*bla*SPM	SPM-F	AAAATCTGGGTACGCAAACG	271	52
	SPM-R	ACATTATCCGCTGGAACAGG		
*bla*GIM	GIM-F	TCGACACACCTTGGTCTGAA	477	50
	GIM-R	AACTTCCAACTTTGCCATGC		
*bla*SIM	SIM-F	TACAAGGGATTCGGCATCG	570	50
	SIM-R	TAATGGCCTGTTCCCATGTG		
*bla*NDM	NDM-F	CACCTCATGTTTGAATTCGCC	984	52
	NDM-R	CTCTGTCACATCGAAATCGC		
blaKPC	KPC-F	CGTCTAGTTCTGCTGTCTTG	798	50
	KPC-R	CTTGTCATCCTTGTTAGGCG		
*arm*A	armA-F	ATTCTGCCTATCCTAATTGG	315	56

	armA-R	ACCTATACTTTATCGTCGTC		
*rmt*A	rmtA-F	CTAGCGTCCATCCTTTCCTC	635	56
	rmtA-R	TTTGCTTCCATGCCCTTGCC		
*rmt*B	rmtB-F	ATGAACATCAACGATGCCCT	769	56
	rmtB-R	CCTTCTGATTGGCTTATCCA		
*rmt*C	rmtC-F	CGAAGAAGTAACAGCCAAAG	711	56
	rmtC-F	ATCCCAACATCTCTCCCACT		
*rmt*D	rmtD-F	CGGCACGCGATTGGGAAGC	401	51
	rmtD-R	CGGAAACGATGCGACGAT		
*rmt*E	rmtE-F	ATGAATATTGATGAAATGGTTGC	818	46
	rmtE-R	TGATTGATTTCCTCCGTTTTTG		
*rmt*F	rmtF-F	GCGATACAGAAAACCGAAGG	589	50
	rmtF-R	ACCAGTCGGCATAGTGCTTT		
aac(6′)-Ib	aac(6')-Ib-F	TTGCGATGCTCTATGAGTGGCTA	482	55
	aac(6')-Ib-R	CTCGAATGCCTGGCGTGTTT		
aph(3′′)-Ib	aph(3'')-Ib-F	GTGGCTTGCCCCGAGGTCATCA	612	55
	aph(3'')-Ib-R	CCAAGTCAGAGGGTCCAATC		
ant(2″)-Ia	ant(2'')-Ia-F	ACGCCGTGGGTCGATGTTTGATGT	572	60
	ant(2'')-Ia-R	CTTTTCCGCCCCGAGTGAGGTG		
qnrA	qnrA-F	GGATGCCAGTTTCGAGGA	492	52
	qnrA-R	TGCCAGGCACAGATCTTG		
qnrB	qnrB-F	GGMATHGAAATTCGCCACTG	264	55
	qnrB-R	TTTGCYGYYCGCCAGTCGAA		
qnrS	qnrS-F	GCAAGTTCATTGAACAGGGT	428	50
	qnrS-R	TCTAAACCGTCGAGTTCGGCG		
qepA	qepA-F	AACTGCTTGAGCCCGTAGAT	596	55
	qepA-R	GTCTACGCCATGGACCTCAC		
*tet*A	tetA-F	GTGAAACCCAACATACCCC	888	50
	tetA-F	GAAGGCAAGCAGGATGTAG		
*tet*B	tetB-F	CCTTATCATGCCAGTCTTGC	774	50
	tetB-R	ACTGCCGTTTTTTCGCC		
*sul*1	sul1-F	CGGCGTGGGCTACCTGAACG	433	58
	sul1-R	GCCGATCGCGTGAAGTTCCG		
*sul*2	sul2-F	GCGCTCAAGGCAGATGGCATT	293	58
	sul2-R	GCGTTTGATACCGGCACCCGT		
traT	traT-F	GGTGTGGTGCGATGAGCACAG	290	58
	traT-R	CACGGTTCAGCCATCCCTGAG		
iutA	iutA-F	GGCTGGACATCATGGGAACTGG	302	58
	IutA-R	CGTCGGGAACGGGTAGAATCG		
hylA	hylA-F	AGCTGCAAGTGCGGGTCTG	569	58
	hylA-R	TACGGGTTATGCCTGCAAGTTCAC		
fyuA	fyuA-F	GTAAACAATCTTCCCGCTCGGCAT	850	58
	fyuA-R	TGACGATTAACGAACCGGAAGGGA		

papC	papC-F	GACGGCTGTACTGCAGGGTGTGGCG	328	62
	papC-R	ATATCCTTTCTGCAGGCAGGGTGTGGC		
papG	papG-F	CTGTAATTACGGAAGTGATTTCTG	1070	50
	papG-R	ACTATCCGGCTCCGGATAAACCAT		
Irp2	Irp2-F	AAGGATTCGCTGTTACCGGAC	413	—
	Irp2-R	AACTCCTGATACAGGTGGC		
capU	capU-F	CAGGCTGTTGCTCAAATGAA	395	52
	capU-R	GTTCGACATCCTTCCTGCTC		
fimH	fimH-F	TGCAGAACGGATAAGCCGTGG	508	58
	fimH-R	GCAGTCACCTGCCCTCCGGTA		
kpsMTII	kpsMTII-R	GCGCATTTGCTGATACTGTTG	272	52
	kpsMTII-F	CATCCAGACGATAAGCATGAGCA		

Table S2: Demographics, collection dates, and hospital ward information of the UTI Patients

No.	Age	Gender	Hospital ward	Collection Date	Hospital
1	64	Female	Gynecology	2/5/2023	A
2	65	Female	Urology	3/5/2023	A
3	81	Male	Urology	7/5/2023	A
4	68	Female	Intensive care unit	29/5/2023	C
5	62	Female	Nephrology	4/6/2023	A
6	66	Male	Intensive care unit	17/6/2023	A
7	67	Male	Intensive care unit	17/6/2023	A
8	57	Male	Nephrology	21/6/2023	A
9	55	Female	Gynecology	29/6/2023	A
10	8	Female	Gynecology	2/7/2023	C
11	68	Female	Gynecology	6/7/2023	A
12	67	Female	Gynecology	18/7/2023	B
13	70	Female	Gynecology	18/7/2023	B
14	71	Female	Intensive care unit	22/7/2023	A
15	55	Female	Intensive care unit	27/7/2023	C
16	65	Female	Nephrology	31/7/2023	A
17	72	Female	Nephrology	31/7/2023	A
18	76	Female	General medicine	1/8/2023	B
19	57	Female	General medicine	3/8/2023	B
20	77	Female	General medicine	6/8/2023	A
21	75	Female	General medicine	6/8/2023	A
22	70	Female	General medicine	9/8/2023	A
23	77	Male	General medicine	9/8/2023	A
24	70	Female	Gynecology	11/8/2023	B
25	83	Male	Intensive care unit	13/8/2023	A
26	61	Female	Intensive care unit	15/8/2023	C
27	71	Female	Surgery	18/8/2023	B
28	76	Female	Gynecology	21/8/2023	A
29	61	Female	Gynecology	21/8/2023	A
30	72	Male	Urology	25/8/2023	A
31	60	Male	Urology	26/8/2023	A
32	67	Female	Intensive care unit	27/8/2023	B
33	73	Male	Nephrology	30/8/2023	C
34	70	Male	Nephrology	30/8/2023	C
35	60	Male	Urology	6/9/2023	A
36	82	Male	General medicine	13/9/2023	C
37	85	Female	General medicine	13/9/2023	C
38	69	Female	General medicine	16/9/2023	C
39	70	Female	Gynecology	17/9/2023	C
40	68	Female	Gynecology	23/9/2023	A
41	62	Male	Surgery	29/9/2023	C
42	70	Female	Surgery	29/9/2023	C
43	54	Female	Intensive care unit	5/10/2023	C
44	93	Female	Intensive care unit	8/10/2023	A
45	65	Male	Surgery	10/10/2023	C
46	82	Female	Gynecology	16/10/2023	A
47	64	Female	Gynecology	17/10/2023	A
48	63	Female	Gynecology	21/10/2023	B

49	75	Female	Gynecology	23/10/2023	C
50	65	Female	Gynecology	25/10/2023	B
51	66	Female	Gynecology	25/10/2023	B
52	80	Male	Intensive care unit	28/10/2023	A
53	70	Female	Nephrology	1/11/2023	A
54	60	Female	Urology	13/11/2023	B
55	65	Female	Surgery	16/11/2023	A
56	60	Female	Gynecology	18/11/2023	B
57	64	Female	Gynecology	21/11/2023	A
58	68	Male	General medicine	23/11/2023	A
59	63	Female	General medicine	23/11/2023	A
60	58	Male	General medicine	24/11/2023	A
61	45	Female	Intensive care unit	25/11/2023	B
62	55	Female	Gynecology	27/11/2023	A
63	55	Male	Intensive care unit	3/12/2023	A
64	45	Female	Urology	5/12/2023	C
65	65	Female	Nephrology	11/12/2023	A
66	60	Male	Urology	13/12/2023	B
67	59	Female	Gynecology	14/12/2023	A
68	45	Female	Gynecology	15/12/2023	B
69	59	Female	Urology	19/12/2023	C
70	55	Female	General medicine	23/12/2023	B
71	59	Female	General medicine	23/12/2023	B
72	60	Male	Nephrology	25/12/2023	A
73	66	Female	Nephrology	27/12/2023	A
74	70	Male	Intensive care unit	29/12/2023	C
75	76	Female	Surgery	2/1/2024	A
76	60	Female	Surgery	3/1/2024	A
77	65	Male	Surgery	5/1/2024	C
78	59	Female	Gynecology	7/1/2024	A
79	72	Female	Gynecology	7/1/2024	A
80	45	Male	Urology	17/1/2024	C
81	45	Female	General medicine	19/1/2024	A
82	67	Male	General medicine	21/1/2024	B
83	40	Female	Surgery	21/1/2024	B
84	60	Male	Intensive care unit	23/1/2024	C
85	80	Female	Gynecology	25/1/2024	B
86	40	Male	Urology	27/1/2024	C
87	72	Female	Intensive care unit	4/2/2024	A
88	16	Female	Intensive care unit	7/2/2024	B
89	60	Female	General medicine	9/2/2024	A
90	30	Female	Gynecology	11/2/2024	B
91	64	Female	Gynecology	13/2/2024	C
92	59	Female	General medicine	16/2/2024	B
93	70	Male	Surgery	16/2/2024	B
94	67	Male	Surgery	17/2/2024	A
95	61	Female	Nephrology	20/2/2024	C
96	66	Female	Gynecology	24/2/2024	B
97	70	Female	Gynecology	24/2/2024	B
98	63	Female	Gynecology	24/2/2024	B
99	68	Female	Intensive care unit	27/2/2024	A

100	35	Female	General medicine	28/2/2024	A
101	60	Female	General medicine	1/3/2024	A
102	62	Female	Gynecology	3/3/2024	B
103	65	Female	General medicine	3/3/2024	B
104	76	Female	Nephrology	4/3/2024	B
105	30	Female	Surgery	6/3/2024	B
106	45	Male	Urology	7/3/2024	A
107	67	Female	General medicine	9/3/2024	A
108	76	Female	Gynecology	9/3/2024	C
109	45	Female	General medicine	9/3/2024	B
110	66	Female	Gynecology	10/3/2024	A
111	62	Female	Gynecology	13/3/2024	A
112	60	Female	General medicine	17/3/2024	A
113	70	Female	Gynecology	19/3/2024	A
114	55	Female	General medicine	21/3/2024	A
115	67	Male	Surgery	23/3/2024	B
116	70	Male	Nephrology	25/3/2024	A
117	40	Male	Nephrology	25/3/2024	A
118	70	Female	Surgery	2/4/2024	C
